# Supplementary material for: Viscosity Prediction of High-Concentration Antibody Solutions with Atomistic Simulations
Source: J Chem Inf Model. 2023 Sep 27;63(19):6129–40. doi: 10.1021/acs.jcim.3c00947 (PMC10565822; doi:10.1021/acs.jcim.3c00947)
Supplement: Supplementary file 1 — ci3c00947_si_001.pdf [file ci3c00947_si_001.pdf]

Supporting Information: Viscosity Prediction of High-Concentration Antibody Solutions with Atomistic Simulations

Tobias M. Prass,[†] Patrick Garidel,[‡] Michaela Blech,[‡] and Lars V. Schäfer[*,†]

†*Center for Theoretical Chemistry, Ruhr University Bochum, D-44780 Bochum, Germany*

‡*Boehringer Ingelheim Pharma GmbH & Co. KG, Innovation Unit, PDB, D-88397 Biberach an der Riss, Germany*

E-mail: lars.schaefer@ruhr-uni-bochum.de

Double- versus tripe-exponential fits

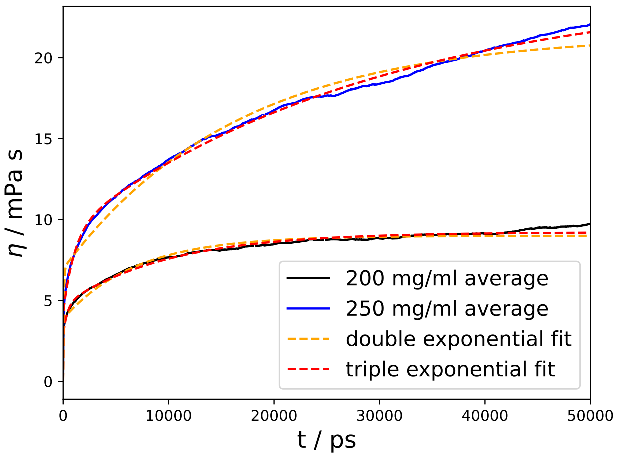

Figure S1: Comparison of a double- and triple-exponential fits to the viscosity curves of the concentrated mAb solutions. The triple-exponential fits, depicted with red dashed lines, capture the actual data at both 200 mg/ml (black curve) and 250 mg/ml (blue curve) more closely than the double-exponential fits (orange dashed lines; the long-time limiting values of the viscosities from the double-exponential fits are $\eta = 9.0$ and $\eta = 21.5$ mPa·s for 200 and 250 mg/ml, respectively).

Viscosity of bulk AMBER a99SB-disp water model

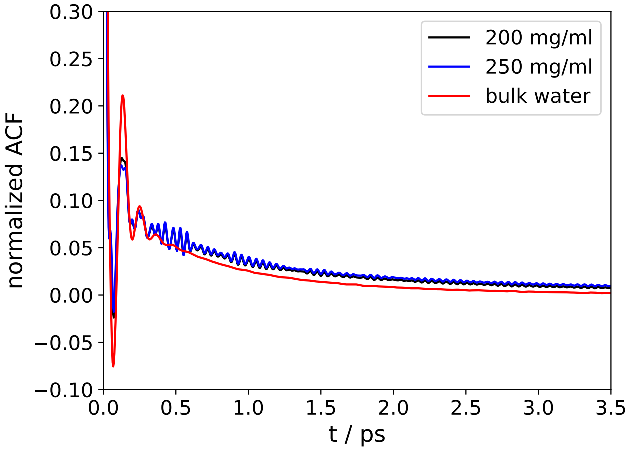

Figure S2: Comparison of the normalized pressure autocorrelation functions (ACFs) between the antibody systems and bulk water (a99SB-disp water model[S1]).

To determine the shear viscosity of bulk a99SB-disp water, a cubic box of 2000 water molecules was simulated in the NpT ensemble at 298 K for 500 ns. Similar to the mAb simulations, 21 configurations taken from the last 400 ns were used to initiate 5 ns NVT simulations during which the pressure was saved to disk every 4 fs. To compute the shear viscosity from the GK integrals, a similar procedure as used for the mAb solutions was followed, with the exception that a double-exponential fit (equation 1)

$$\eta(t) = B \cdot \alpha \cdot \tau_1 \left(1 - e^{-t/\tau_1}\right) + B \cdot (1-\alpha) \cdot \tau_2 \left(1 - e^{-t/\tau_2}\right) \tag{1}$$

with fitting parameters $B > 0$, $\alpha, \beta < 1$, and $\tau_{1,2} > 0$, was used to extrapolate the GK integrals to the long time limit.

The 21 viscosity graphs and average are shown in Figure S7. The viscosity of bulk a99SB-disp water obtained from the fit to the average of the 21 GK runnning integrals is 1.07 mPa·s, which is slightly higher than the value of 1.02 mPa· reported by Qiu et al. (2021),[S2] who estimated the viscosity from the self-diffusion coefficient. The a99SB-*disp* water model thus overestimates the experimental value of 0.896 mPa·s at 25 °C[S3] by ca. 20%.

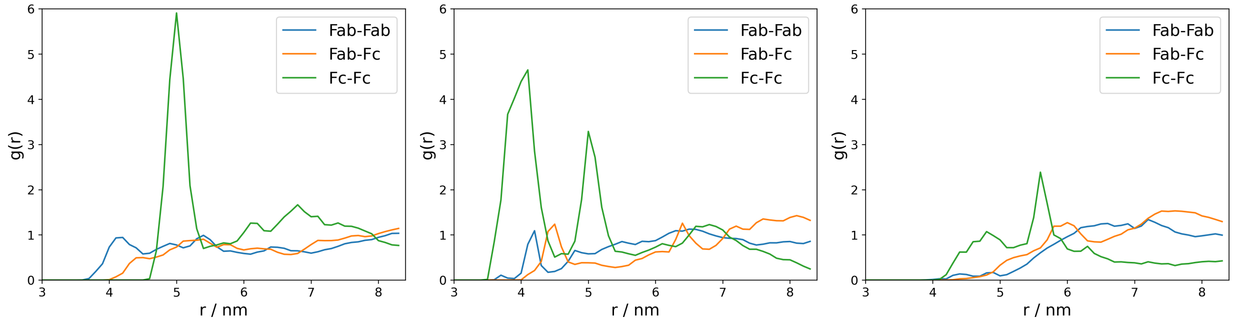

1 2 3

Figure S3: Radial distribution functions of the antibody domains with respect to the domain centers-of-mass in the 200 mg/ml simulation sets 1, 2 and 3.

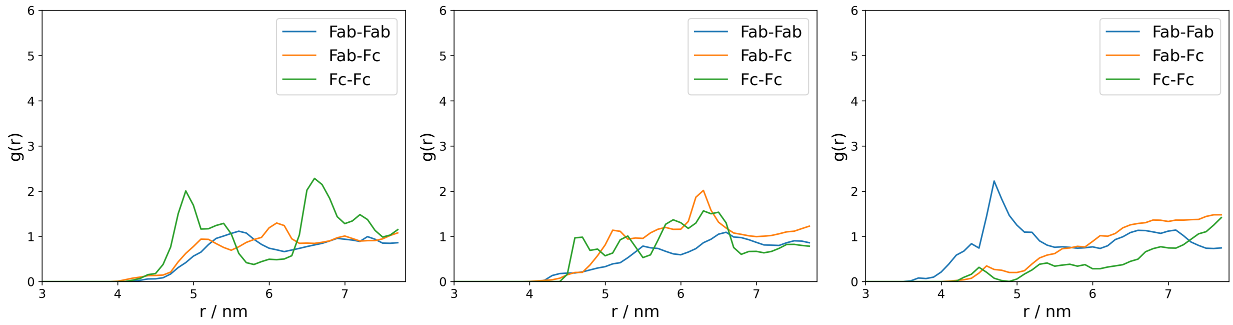

1 2 3

Figure S4: Radial distribution functions of the antibody domains with respect to the domain centers-of-mass in the 250 mg/ml simulation sets 1, 2 and 3.

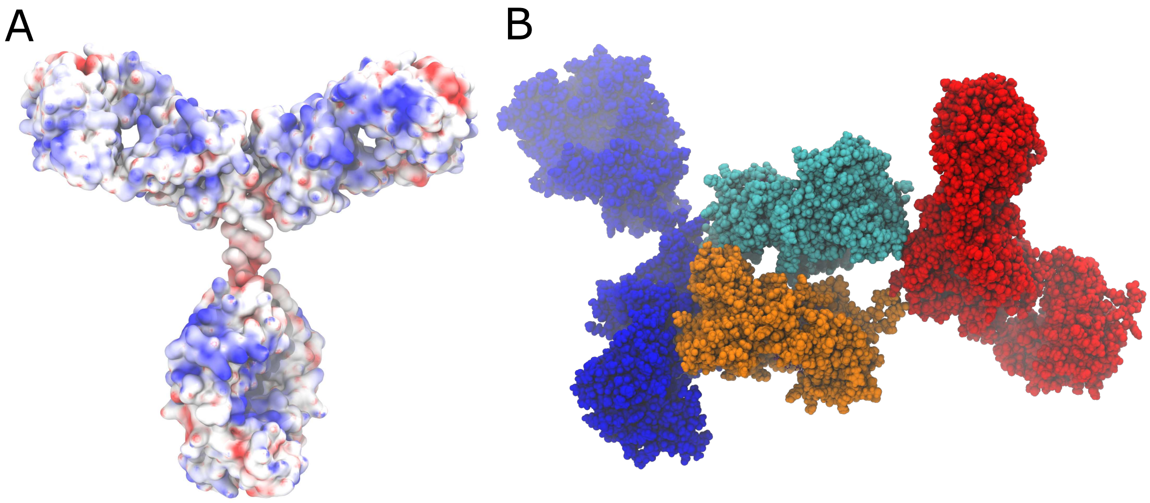

Figure S5: A) Visualization of the electrostatic potential mapped onto the mAb surface. B) Snapshot taken from "set 2" of the 200 mg/ml simulations, showing 2 of the mAbs forming a dimer via the Fc domains (colored in cyan and orange).

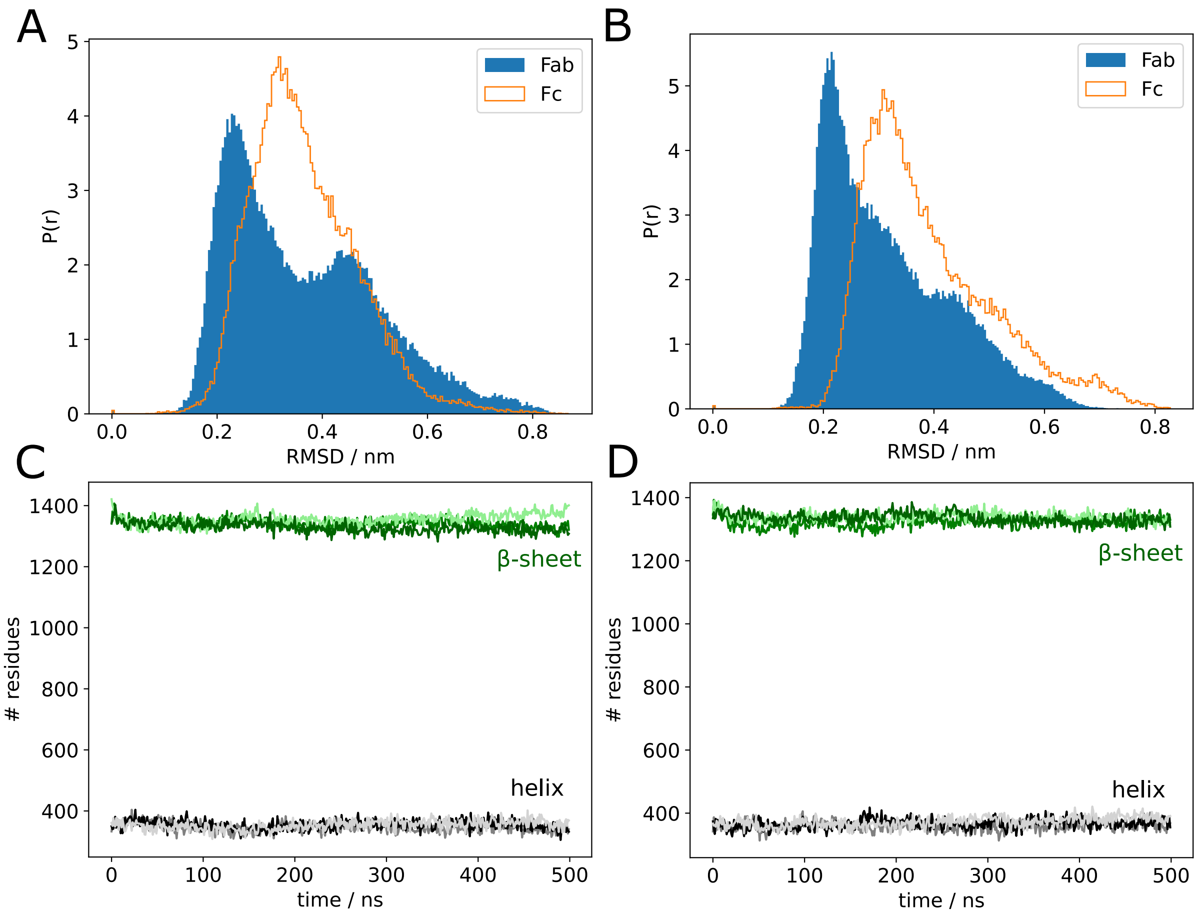

Figure S6: Analysis of the structural integrity of the mAbs in the simulations. A) and B) show the Cα-RMSD distributions in the 200 mg/ml and 250 mg/ml mAb solutions, respectively. C) and D) show the time evolution of the number of residues in β-sheets and helices in the 200 mg/ml and 250 mg/ml systems, respectively. This residue count is over all 4 mAbs in the simulation systems.

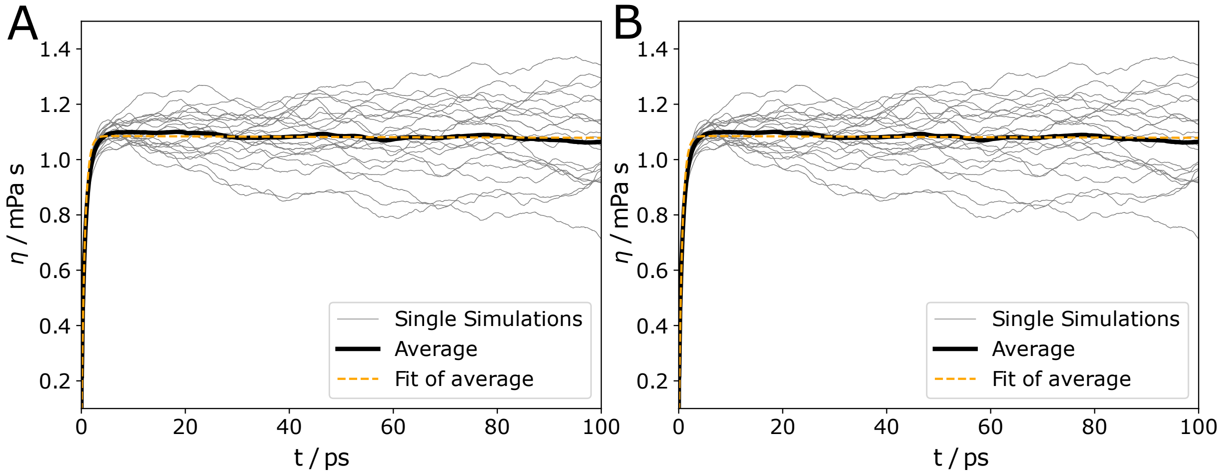

Figure S7: Running GK integrals from 21 individual MD trajectories of bulk water (a99SB-disp water model), computed in with integration time steps of A) 2 fs and B) 4 fs. The individual integrals are plotted as thin grey lines, the average is plotted as a thick black line. The dashed lines show the double-exponential fits, $\eta(t) = B \cdot \alpha \cdot \tau_1 \left(1 - e^{-t/\tau_1}\right) + B \cdot (1 - \alpha) \cdot \tau_2 \left(1 - e^{-t/\tau_2}\right)$, which yield long-time limit viscosities of $\eta = 1.0757$ and 1.0745 mPa·s for the simulations with the 2 fs and 4 fs integration time steps, respectively.

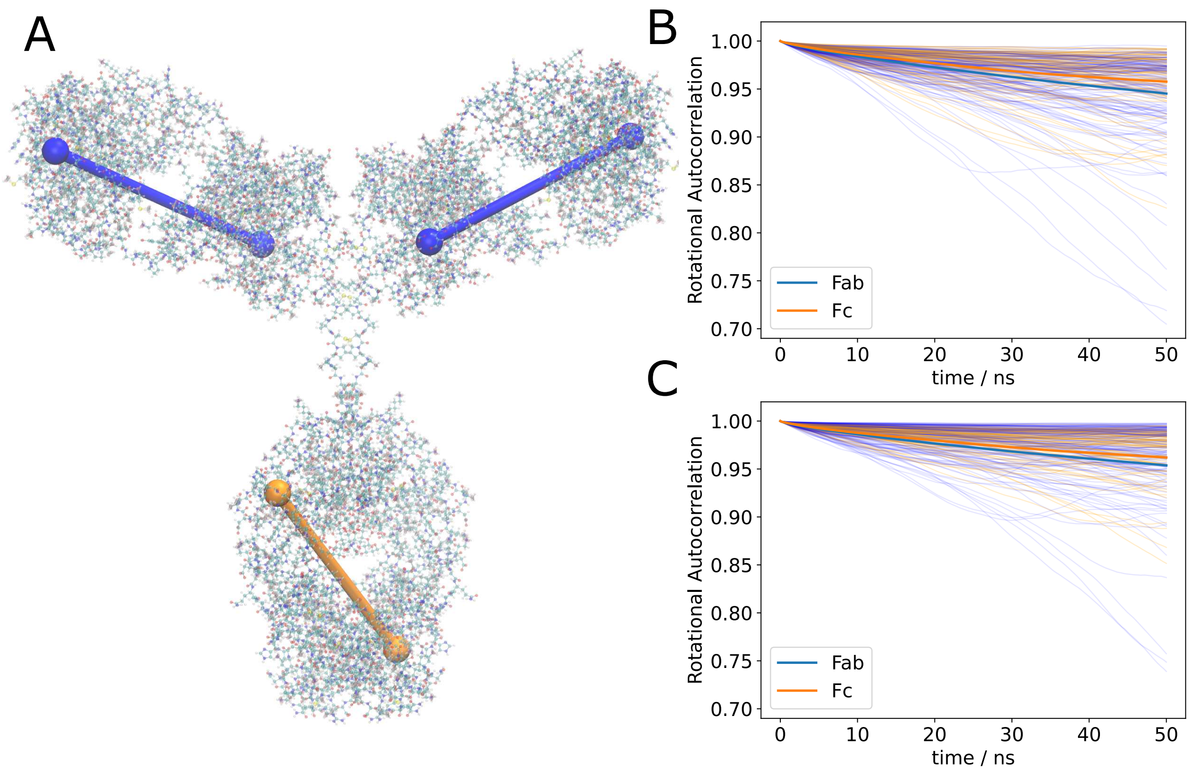

Figure S8: Time correlation functions (TCFs) describing the reorientation motions (orientational diffusion) of the mAb domains. Here, the TCF of the cosine of the angle α is plotted, where α is the angle between a vector at time t_0 and at time $t_0 + t$. A) shows the analyzed vectors that span across the individual mAb domains (Fab: LC Ala72 to HC Cys209 (blue); Fc: HC1 Trp286 to HC2 Val357 (orange)). B) and C) show the TCFs of the Fab (blue) and Fc (orange) domains in the 200 mg/ml and 250 mg/ml systems, respectively.

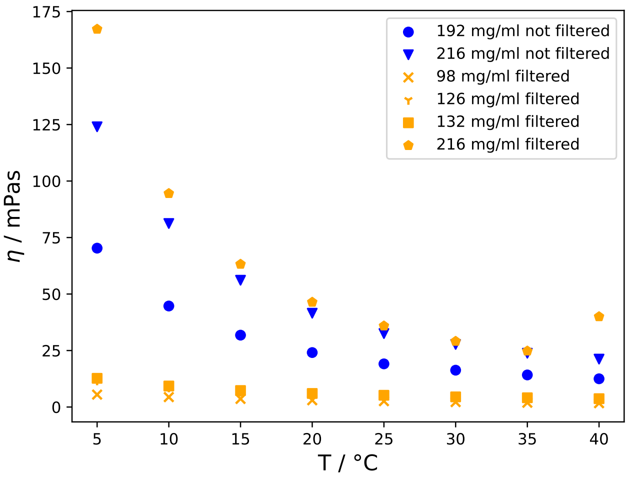

Figure S9: Rotational rheometry viscosities from the temperature ramps. The data from the filtered and unfiltered samples are depicted in orange and blue, respectively.

References

(S1) Robustelli, P.; Piana, S.; Shaw, D. E. Developing a molecular dynamics force field for both folded and disordered protein states. *Proc. Natl. Acad. Sci. U.S.A.* **2018**, *115*, E4758–E4766.

(S2) Qiu, Y.; Jiang, Y.; Zhang, Y.; Zhang, H. Rational Design of Nonbonded Point Charge Models for Monovalent Ions with Lennard-Jones 12-6 Potential. *J. Phys. Chem. B* **2021**, *125*, 13502–13518.

(S3) Harris, K. R.; Woolf, L. A. Temperature and Volume Dependence of the Viscosity of Water and Heavy Water at Low Temperatures. *J. Chem. Eng. Data* **2004**, *49*, 1064–1069.
